# Nutritional composition of *Zea mays L*: A comprehensive Review of Macronutrients, Micronutrients, and Bioactive Compounds

**DOI:** 10.12688/openresafrica.16056.2

**Published:** 2026-02-13

**Authors:** Sandra Etumah Ifie, Reuben Samson Dangana, Dominic Swase, Michael Ben Okon, Wusa Makena, Mary Olaoluwa Agunloye, Chinyere Nneoma Ugwu, Josiah Eseoghene Ifie, Saidi Odoma, Abubaka Ibrahim Babangida, Patrick Maduabuchi Aja

**Affiliations:** 1 Biochemistry, Kampala International University - Western Campus, Bushenyi, Western Region, Uganda; 2 Genetics, University of KwaZulu-Natal - Westville Campus, Durban, KwaZulu-Natal, South Africa; 3 Human Anatomy, Kampala International University - Western Campus, Bushenyi, Western Region, Uganda; 4 Physiology, Kampala International University - Western Campus, Bushenyi, Western Region, Uganda; 5 Publication and Extension, Kampala International University - Western Campus, Bushenyi, Western Region, Uganda; 6 Department of Science, Valley University of Science and Technology, Bushenyi, Uganda; 7 Pharmacology, Kampala International University - Western Campus, Bushenyi, Western Region, Uganda

**Keywords:** Zea mays, nutritional composition, biofortification, micronutrient deficiencies, functional food

## Abstract

**Background:**

Maize ( *Zea mays L.*) is a significant cereal crop, which, due to its nutritious content, especially in terms of dietary energy and nutrients, is globally important. Its nutritive status does not just end with macronutrients, but it also encompasses nutrient status that promotes health-giving micronutrients and phytochemicals.

**Objective:**

This review aims to consolidate recent advancements in maize nutrient profiling and highlight current challenges in optimizing its nutritional potential across global food systems.

**Methods:**

A comprehensive literature search was conducted in PubMed, Scopus, and Google Scholar for peer-reviewed articles published between 2010 and 2024 in English. Search terms included “Zea mays nutrition,” “maize macronutrients,” “maize micronutrient biofortification,” and “phytochemicals in maize”. Studies included both empirical and review papers reporting on maize nutritional quality, bioavailability, and impacts on human health.

**Results:**

Maize is composed primarily of carbohydrates, with starch as the dominant fraction, alongside moderate protein and low lipid content concentrated in the germ. Biofortified varieties, including Quality Protein Maize (QPM), provide enhanced levels of lysine, tryptophan, and provitamin A. Maize also supplies essential B vitamins and minerals such as iron, zinc, and magnesium; however, their bioavailability is reduced by antinutrients like phytates. In addition, maize is shown to be rich in phytochemicals, including ferulic acid, flavonoids, and carotenoids, which exhibit antioxidant and therapeutic properties.

**Conclusion:**

Maize is more than a staple food crop; it is an indispensable crop for global food and nutrition security. However, challenges such as low bioavailability due to antinutritional factors limit its full nutritional potential. Therefore, strengthening genetic and agronomic interventions to improve its nutritional quality is essential to address hidden hunger and enhance population health worldwide.

## Introduction

Maize ( *Zea mays L.*), or common corn, is one of the most respected agricultural and nutritional pillars in the world's cosmos. Commenting on its domestication process put around 20,000 B.C. in Mesoamerica ( García-Lara & Serna-Saldivar, 2019; Téllez-Pérez *et al.*, 2021), maize has transitioned from being part of one or another ancient form of subsistence crop to becoming one of the major food crops for humans. Other than simple consumption, medicine, research, pharmaceuticals, and industries in themselves act as avenues through which maize exhibits human multifaceted existence ( Adiaha, 2017). It remains the firstly produced cereal in the world and the third most consumed after wheat and rice ( FAO, 2021; Galani *et al.*, 2022). These three staples furnish around 42% of the world's calories and 37% of protein ( Erenstein *et al.*, 2022; FAOSTAT, 2021). Maize is cultivated extensively across all agro-ecological zones which demonstrates its remarkable adaptability to diverse climates and soil types. This ecological flexibility not only supports its widespread cultivation but also enables the development of region specific maize varieties tailored to local and global environmental conditions ( Adiaha, 2017). Maize production is predominantly carried out by smallholder farmers under rain-fed conditions, making it highly sensitive to climatic variability and resource constraints ( Egah *et al.*, 2024).

Production in Sub-Saharan Africa alone accounts for 40% of cereal production, over 80% conversion to food, feed, or industrial usage ( FAOSTAT, 2016). Significantly, over 300 million people in Africa consume maize as a staple ( Galani *et al.*, 2022; Mathenge *et al.*, 2014). Nutritionally, maize provides about 365 kcal per 100 g and comprises primarily of 72% starch, 10% protein, 4% fat, considerable amounts of fiber, sugar, and minerals ( Huma *et al.*, 2019; Mulyati *et al.*, 2021). In addition to its consumption as whole grains, maize ( *Zea mays L.*) is widely processed into a variety of food products, which includes cornmeal, tortillas, flatbreads, starches, and breakfast cereals. These products reflect the functional diversity of maize and its central role in both traditional and industrial food systems ( Mehta & Dias, 1999; Tajamul *et al.*, 2016). The nutraceutical potential of maize is a modern concern with maize. As a functional food, maize provides macronutrients along with bioactive compounds like, carotenoids, phenolic acids, flavonoids, phytosterols, and lignins ( Sheng *et al.*, 2018), which act as antioxidants and provide disease-preventive actions, Likewise the chances of contracting disease like diabetes, cardiovascular problems, obesity, or digestive disorders are degreased by Regular intake of such foods ( Baranowska, 2023). Generally, the conventional variants of maize, amongst others, include iron, zinc, and provitamin A compounds, but lack the nutritional aspects most needed by populations who rely on maize for food consumption. Another term for this dilemma is "hidden hunger," which impacts women and children the most ( Galani *et al.*, 2022). Addressing this need, breeding efforts have brought to life varieties of Quality Protein Maize (QPM) and provitamin A cultivars to enhance amino acid and micronutrient profiles.

Nutritional profiling in maize is a systematic evaluation of the nutritional content of maize, and this has presently become instrumental in informing breeding programs, dietary guidelines, and agricultural policies ( Ekpa *et al.*, 2018). It arms school feeding programs, food aid strategies, and other initiatives designed to combat malnutrition through nutrient-sensitive agriculture. According to FAO (2020) and Galani *et al.* (2022), maize production hit a record of more than 1 billion tonnes worldwide in 2016; hence, the sheer potential maize has in influencing food systems, livelihoods, and health outcomes is Incomparable, additionally, this also suggests of maize economic values in alleviating poverty in developing countries like Africa and sub-Saharan Africa. Therefore, this narrative review brings into the latest insights related to the nutrient contents of *Zea mays L.* concerning the crop's macronutrient, micronutrient, and phytochemical potentials, and the food aspect is emphasized. While also reviewing the beneficial effects observed on human health and therefore also scientific interventions toward realizing the full nutritional potential of maize, it is thus discussed as a food security crop.

## Methodology

### Research design

This review employed a narrative approach to synthesize existing literature and identify gaps on the nutritional composition of *Zea mays L.*

### Literature search strategy

A comprehensive search was conducted across multiple scientific databases, including PubMed, Scopus, Web of Science, and Google Scholar, focusing on peer-reviewed literature published between 2010 and 2024. The following search terms were used: *"Zea mays nutrition"*, *"maize bioactive compounds"*, *"micronutrient profile of maize"*, *"QPM maize"*, *"maize phytochemicals"*, and *"biofortified maize varieties".*

### Data collection methods

The collected data were thematically grouped into macronutrient content, micronutrient density, phytochemical presence, and varietal differences based on processing, genotype, and environment. Relevant references were selected for their methodological rigor and relevance to human nutrition.

### Inclusion criteria

Studies were included based on the following criteria:

• Original research articles and review papers
• Studies in English
• Works reporting macronutrient, micronutrient, or bioactive content in maize
• Research on bioavailability and health implications

### Exclusion criteria

• Non-peer-reviewed sources
• Duplicates and short communications without sufficient data
• Studies focusing only on agronomic yield without nutritional analysis

## Macronutrient composition

Maize ( *Zea mays L.*), a key crop in the world's agriculture and nutrition, is desired for its high content of macronutrients such as carbohydrates, proteins, and lipids. These macronutrients vary greatly between maize varieties, e.g., macronutrients present in dent corn, flint corn, popcorn, Quality Protein Maize (QPM), sweet corn, and plant parts (kernels, silk, husk), which determine their nutritive and industrial usability. The nutritional value of maize is the capability to provide energy, growth and repair, and efficient body health due to the composition of its diverse macronutrients. This section will discuss the carbohydrate composition such as the starch types and dietary Fibre, the protein content and amino acid profile as well as the lipid content and fatty acid profile and the associated nutritional importance and variability related to this content based on some recent studies ( Dada *et al.*, 2023; Joshi *et al.*, 2022; Mandal *et al.*, 2023; Swati *et al.*, 2024).

### Carbohydrates: Starch types and dietary fibre

The macronutrient composition of maize is characterized by carbohydrates that are predominantly present as starch and make up the vast majority of the endosperm in the kernel, and act as a major source of energy in diets across the world. Maize starch consists of two primary polysaccharides, which are amylose, i.e., a linear polymer, and amylopectin, i.e., a branched polymer, and the proportion of the two differs according to the type of maize. An example is QPM, which has 24.82–25.11% amylose and 74.30–75.18% amylopectin contents and has an A-type X-ray diffraction pattern that makes it more easily digestible than normal maize ( Joshi *et al.*, 2022). This structural characteristic makes QPM starch more rapidly digestible by amylases, benefiting populations reliant on maize as a staple for energy provision. In contrast, waxy corn, a variety with high amylopectin content (nearly 100%), is prized for its sticky texture in food applications, while flint and dent corn, with balanced amylose-amylopectin ratios, are suited for both food and industrial uses ( Mandal *et al.*, 2023). Beyond the kernel, other maize parts like corn silk, husk, and tassels are rich in carbohydrates, often overlooked as by-products. Corn silk and husk, for example, contain significant carbohydrate content, contributing to their potential as functional food ingredients ( Swati *et al.*, 2024). In popcorn ( *Zea mays L.* var. everta), starch accounts for approximately 47.50% of the kernel's composition, supporting its role as an energy-dense snack food ( Dada *et al.*, 2023). The carbohydrate profile of maize is further enhanced by processing techniques, such as extrusion and fermentation, which can modify starch structure to improve digestibility and glycaemic response, making maize products like tortillas and porridges more nutritionally accessible ( Joshi *et al.*, 2022).

Dietary Fibre, another critical carbohydrate component, is concentrated in the maize seed coat and by-products like corn silk and husk. Fibre in maize primarily consists of hemicellulose (approximately 6%), cellulose (2%), and lignin (0.1%) in the seed coat, with smaller amounts in the endosperm and germ ( Mandal *et al.*, 2023). In popcorn, dietary Fibre content is reported at 3.38%, with neutral detergent Fibre (NDF) at 27.10% and acid detergent Fibre (ADF) at 16.96%, particularly higher in plots treated with inorganic fertilisers like NPK 20-7-3 ( Dada *et al.*, 2023). Fibre contributes to digestive health, regulates blood sugar levels, and supports cardiovascular health by reducing cholesterol absorption. Corn silk and husk, often discarded during processing, are valuable Fibre sources, with potential applications in functional foods and nutraceuticals ( Swati *et al.*, 2024). The underutilization of these by-products highlights an opportunity to enhance the nutritional value of maize-based diets, particularly in regions where dietary Fibre intake is suboptimal.

### Protein content and amino acid profile

Proteins in maize are essential for growth, tissue repair, and metabolic functions, though their content and quality vary across varieties and cultivation practices. Standard maize varieties, such as dent corn, flint corn, and popcorn, typically contain moderate protein levels, ranging from 8–16% of kernel dry weight. Popcorn, for instance, has a protein content of 16.20%, with higher levels observed in NPK-fertilised plots due to the rapid availability of nitrogen compared to organic fertilisers like composted chicken manure, which exhibit slower mineralisation ( Dada *et al.*, 2023). However, the protein quality of conventional maize is limited by its low levels of essential amino acids, particularly lysine and tryptophan, which restricts its biological value to approximately 45% ( Joshi *et al.*, 2022).

QPM addresses this limitation through genetic modifications that enhance the levels of lysine (2.6–4.8% of protein) and tryptophan (0.68–1.8% of protein), resulting in a biological value of 70%, significantly higher than that of normal maize ( Joshi *et al.*, 2022; Mandal *et al.*, 2023). These essential amino acids play a pivotal role in human nutrition, augmenting protein production and metabolic processes, and thus QPM would be an effective resource in the fight against the global-health problem of protein-energy malnutrition disorders such as kwashiorkor in developing nations. The varieties of QPM bear a better profile in other essential amino acids like isoleucine, threonine, globulin, and albumin, such as Vivek QPM9 and Pusa HM4, which further enhance nutritional content ( Joshi *et al.*, 2022). Corn silk and husk also contribute to the protein content of maize, offering a supplementary source of this macronutrient that is often discarded during processing ( Swati *et al.*, 2024).

Zeins has been shown to be the main protein component found in maize apart from lysine and tryptophan and this limit the biological value of maize ( Vasal, 2000). In population where maize is a staple food, this deficiency has significant nutritional significance and needs to be addressed. One of the key strategies to address this is the use of bio-fortification for improved nutritional quality especially in *Zea mays* L. ( Bouis & Saltzman, 2017). Among the most successful examples of the use of the bio-fortification strategy is the Quality Protein Maize (QPM), which was developed through the incorporation of the opaque-2 gene and endosperm modifiers in order to increase the lysine and tryptophan levels while maintaining kernel hardness ( Vasal, 2000). When compared to conventional maize, the QPM varieties showed that the content of zein was reduced while the non-zein proteins, were increased; resulting in nearly twice the contents of lysine and tryptophan ( Alamerew, 2008; Paul *et al.*, 2023). The improvement enhance the biological value of maize protein significantly and have been associated with favorable nitrogen utilization and as well as improved growth outcomes in children. This has underscore the roles of bio-fortification in strengthening the macronutrient quality of maize ( Gupta *et al.*, 2009; Joshi *et al.*, 2022; Prasanna *et al.*, 2001). Bio-fortification projects have not only improved the quality of proteins, but they have also created maize varieties that are high in pro-vitamin A and iron and zinc, which has helped to address micronutrient deficiencies in at-risk groups ( Bouis *et al.*, 2011; Pixley *et al.*, 2019). These improvements have shown how traditional and contemporary plant breeding methods may be used to enhance the macronutrient profiles of maize in a planned way ( Saltzman *et al.*, 2013).

Maize products are processed through methods like extrusion and fermentation that improve the digestibility of proteins and the availability of amino acids in the products. Take an instance of QPM-based tempeh and instant flours, which have higher quality proteins, since they are produced through these methods, and are therefore applicable to other food elements such as biscuits, muffins, and non-alcoholic beverages ( Joshi *et al.*, 2022). The influence of nitrogen sources on protein content, as seen in popcorn studies, underscores the importance of agricultural practices in optimising maize's nutritional value ( Dada *et al.*, 2023). By prioritising biofortified varieties and innovative processing, maize can serve as a high-quality protein source in global diets.

### Lipids: Oil content and fatty acid composition

Though lipids are only available in low ratios relative to carbohydrates and proteins, they are a vital constituent of maize, and in the germ of the kernel, oil is highly concentrated. In conventional maize varieties, the oil content typically ranges between 3–5%, but in some hybrids and biofortified lines, it can reach up to about 7% ( Mandal *et al.*, 2023). Maize lipid profile shows a favourable fatty acid composition, such as the polyunsaturated fatty acids (PUFA), monounsaturated fatty acids (MUFA), and saturated fatty acids. An example of this is popcorn, which contains PUFA and MUFA as important elements that help in the nutritional value of the kernels ( Dada *et al.*, 2023). Maize oil is rich in linoleic acid (a PUFA, 50–60%), oleic acid (a MUFA, 25–35%), and smaller amounts of palmitic and stearic acids (saturated fatty acids, 10 – 15%), making it a heart-healthy oil with applications in cooking and food processing ( Mandal *et al.*, 2023).

Maize oil contains fatty acids in genotype-dependent and environment-dependent proportions. As an example, breeding biofortified maize varieties might result in changes in lipid profile because of nutritional enhancement breeding. QPM and other high-oil maize lines may possess slightly elevated oil content with a balanced proportion of PUFA to MUFA that promotes cardiovascular fitness by lowering levels of low-density lipoprotein (LDL) cholesterol ( Joshi *et al.*, 2022). Also, maize oil contains phytochemicals, plant sterols, like stigmasterol, other than cholesterol, sitosterol, and campesterol, which are used in lowering cholesterol ( Mandal *et al.*, 2023). Such compounds increase the functional food capability of maize oil, especially when used in processed foods such as margarine and salad dressings. The lipids are present as trace compounds in the corn silk and corn husk, but all these are insignificant compared to the kernel germ ( Swati *et al.*, 2024). Non-chemical lipid preservation methods protect the lipid content of maize, avoiding the process of oxidative rancidity and keeping nutritional value after harvest by using filter cake powder and Purdue Improved Crop Storage bags ( Mandal *et al.*, 2023). Maize oil belongs to the family of fats rich in unsaturated fatty acids, with the highest proportion of about 50–60% being omega-6 ( Mandal *et al.*, 2023). This makes it a very useful dietary and industrial ingredient with significant potential to comprise the macronutrient content of the diet, it is a well-known phytochemical component in maize oil, which is the most plentiful member of the maize species.

The nutritional value of maize derives from its functionality and nutritional value in the macronutrient content of its carbohydrates, proteins, and lipids. Starch is the main carbohydrate and is also a dependable source of energy, and dietary Fibre can increase the health values, especially in the lesser-exploited by-products such as corn silk and husk. Proteins, particularly in QPM, provide better balanced amino acids to overcome the nutritional deficiency, and lipids, especially concentrated in germ, provide the heart-friendly fats and phytochemicals. The measured variations of macronutrient concentrations in maize cultivars and the existence of agricultural processing factors contribute to the potential to optimise the nutritional content of maize to address dietary energy requirements on a global scale ( Dada *et al.*, 2023; Joshi *et al.*, 2022; Mandal *et al.*, 2023; Swati *et al.*, 2024). Table 1 summarises the macronutrient composition of various maize types and parts, highlighting the variability in carbohydrates, proteins, and lipids across studies.

**Table 1.** Macronutrient Composition of Maize ( *Zea mays L.*) Across Varieties and Plant Parts.

S/No	Macronutrient	Component	Maize Type/Part	Composition	Reference
1	Carbohydrates	Starch	Quality Protein Maize (QPM)	Amylose: 24.82–25.11%, Amylopectin: 74.30–75.18%; A-type X-ray diffraction pattern, highly digestible	( Joshi *et al.*, 2022)
			Popcorn ( *Zea mays L.* var. everta)	Starch: 47.50%	( Dada *et al.*, 2023)
			Flint Dent Flour Corn	High starch content in endosperm, primarily amylose and amylopectin (specific ratios not provided)	( Mandal *et al.*, 2023)
			Waxy Corn	Nearly 100% amylopectin	( Mandal *et al.*, 2023)
			Corn Silk Husk Tassels	Significant carbohydrate content is underutilized	( Swati *et al.*, 2024)
		Dietary Fibre	Popcorn	Fibre: 3.38%, Neutral Detergent Fibre (NDF): 27.10%, Acid Detergent Fibre (ADF): 16.96%	( Dada *et al.*, 2023)
			Flint Popcorn Flour Corn	Seed coat: ~6% hemicellulose, 2% cellulose, 0.1% lignin; lower in endosperm and germ	( Mandal *et al.*, 2023)
			Corn Silk Husk	High fibre content is valuable for functional foods	( Swati *et al.*, 2024)
9	Proteins	Total Protein	Popcorn	16.20% higher in NPK-fertilised plots	( Dada *et al.*, 2023)
			Quality Protein Maize (QPM)	8–16% (varies by variety), biological value 70% (vs. 45% for normal maize)	( Joshi *et al.*, 2022)
			Dent Flint Sweet Corn	Moderate protein content (8–16%), lower biological value	( Mandal *et al.*, 2023; Swati *et al.*, 2024)
			Corn Silk Husk	Present supplementary protein source	( Swati *et al.*, 2024)
		Amino Acid Profile	Quality Protein Maize (QPM)	Lysine: 2.6–4.8% of protein (~0.07 g/100 g starch), Tryptophan: 0.68–1.8% of protein; higher isoleucine, threonine, globulin, albumin	( Joshi *et al.*, 2022; Mandal *et al.*, 2023)
			Normal Maize (e.g. Flint Dent)	Low lysine and tryptophan limit nutritional quality	( Joshi *et al.*, 2022)
15	Lipids	Oil Content	Flint Popcorn Flour Corn	3–5% in kernels, up to 7% in high-oil hybrids; concentrated in the germ	( Mandal *et al.*, 2023)
			Corn Silk Husk	Trace amounts	( Swati *et al.*, 2024)
		Fatty Acid Composition	Popcorn	High in polyunsaturated fatty acids (PUFA) and monounsaturated fatty acids (MUFA)	( Dada *et al.*, 2023)
			Flint Popcorn QPM	Linoleic acid (PUFA): 50–60%, Oleic acid (MUFA): 25–35%, Palmitic/Stearic acids (Saturated): 10–15%; contains plant sterols (stigmasterol, sitosterol, campesterol)	( Mandal *et al.*, 2023)

**Legend:** This table illustrates the proximate composition of maize, including carbohydrates, proteins, lipids, and dietary fiber, highlighting its macronutrient profile.

## Micronutrient profile of *Zea mays L.*

**Figure 1.**
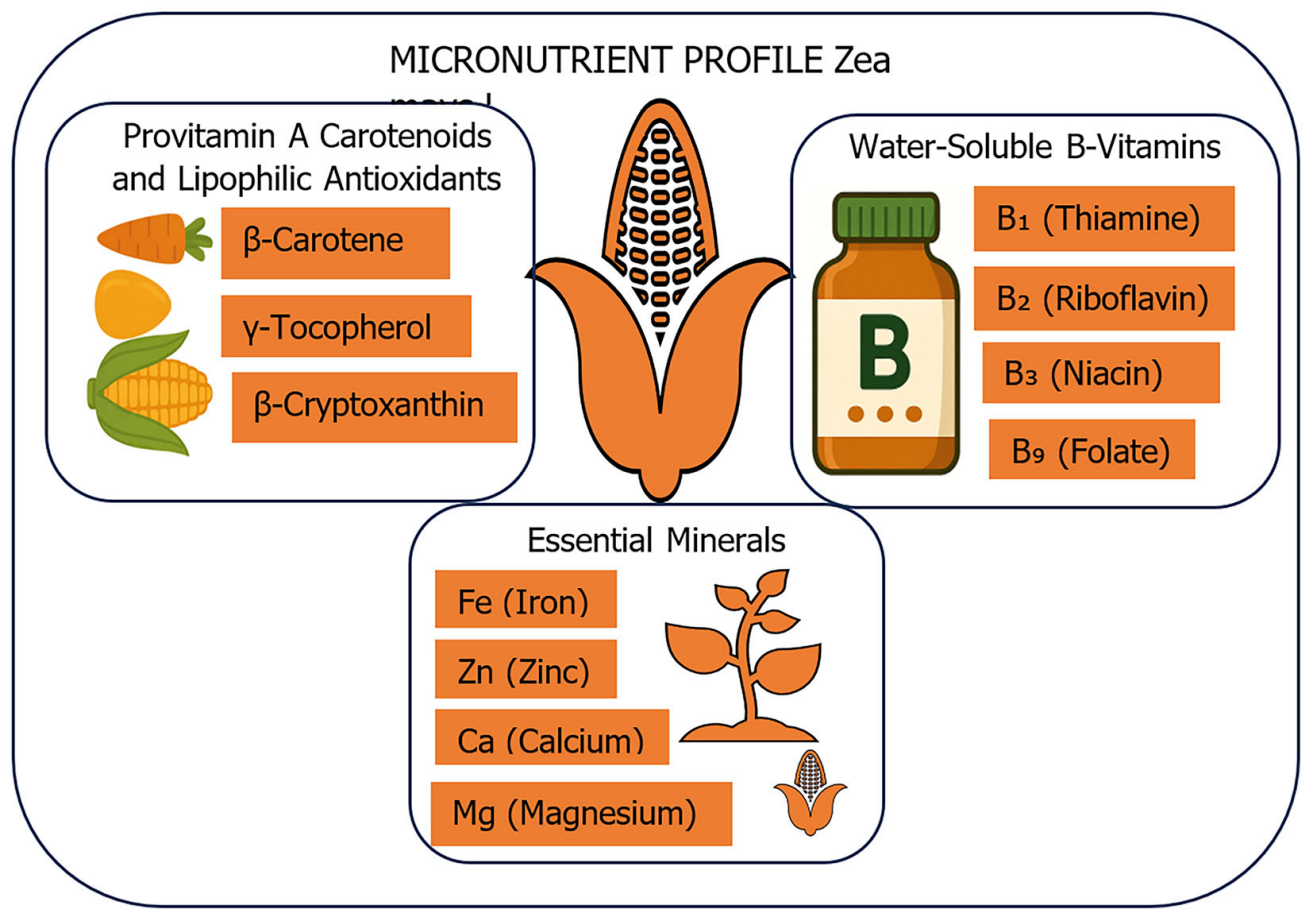
Micronutrient Composition of *Zea mays L.*: Key Vitamins and Minerals. The figure illustrates the key micronutrients found in *Zea mays L.* (maize), including provitamin A carotenoids (β-carotene, β-cryptoxanthin), lipophilic antioxidants (γ-tocopherol), essential water-soluble B-vitamins (B _1_, B _2_, B _3_, B _4_), and critical minerals (iron, zinc, magnesium, and calcium) vital for metabolic function, immune health, and nutritional adequacy in maize-based diets.

Maize ( *Zea mays L.*) is valued worldwide for its carbohydrate and energy levels. It plays a key role in providing essential micronutrients that support the body's biochemical and physiological functions. The micronutrient density and bioavailability in maize are affected by factors such as genotype, soil conditions, post-harvest processing, and the presence of anti-nutritional compounds, including phytates ( Amanjyoti *et al.*, 2024). The micronutrient profile of *Zea mays L.* can be divided into three main groups: provitamins and antioxidants, B-complex vitamins, and essential minerals. This classification reflects their functional roles, bioaccessibility, and overall nutritional importance as shown in Figure 1.

### Provitamins and antioxidants

*Zea mays L.* contains high levels of carotenoids, including β-carotene, β-cryptoxanthin, and α-carotene, which are important for nutrition. These compounds are precursors of retinol, the active form of vitamin A responsible for visual function, epithelial integrity, and immune defense. However, most conventional yellow maize varieties contain insufficient quantities of these compounds to meet daily requirements ( Beer *et al.*, 2024). This micronutrient gap is of particular concern in sub-Saharan Africa and South Asia, where vitamin A deficiency remains a leading cause of blindness and increased morbidity among children and pregnant women. This has led to the development of biofortified orange maize to deliver sufficient levels of provitamin A to meet public health demand ( Arlappa *et al.*, 2024). It has been shown that foods that contain orange maize have resulted in elevated serum retinol levels following consumption of these foods. Also, it has been reported to lower the prevalence of subclinical deficiency of vitamin A among reproductive age women and children ( Afolami *et al.*, 2021). These findings indicate the practical nutritional utility of provitamin A-enriched maize in regions where dietary diversification is limited. In addition to carotenoids, maize germ provides sufficient amounts of vitamin E in the form of tocopherols and tocotrienols. Maize is rich in γ-tocopherol, which has been reported by recent research as an important lipid-soluble antioxidant ( Bello *et al.*, 2021). Also, Vitamin E protects polyunsaturated fatty acids in cell membranes against oxidative degradation and regulates the inflammatory signaling, which it does by inhibiting protein kinase C. Maize oil, especially that made by high-oil hybrids, is rated as a good dietary source of tocopherols, with a concentration between 100 to 180 mg/kg. Such antioxidants have also been associated with lower cardiovascular disease and neurodegeneration risks, implying the potential health ramifications of the lipid-soluble vitamins found in maize ( Ungurianu *et al.*, 2021).

### B-vitamins and metabolic cofactors

Maize also contains important water-soluble B-vitamins, which serve as metabolic cofactors and are required for energy metabolism, redox control, and neurodevelopment. Key B-complex vitamins found in maize include thiamine (B1), riboflavin (B2), niacin (B3), and folate is present in different concentrations in maize kernels. Among these, niacin exists predominantly in a chemically bound form known as niacytin, which is poorly bioavailable in its native state ( Hrubša *et al.*, 2022). Niacin is present in unprocessed forms, like nixtamalization (alkaline cooking) in which niacin is unavailable to the human digestive system. Historically, this biochemical deficiency led to the incredible prevalence of pellagra, a debilitating deficiency disorder that is characterized by the following triple Ds: dermatitis, diarrhea, and dementia ( Malik *et al.*, 2023). These conditions have been reported to be particularly prevalent in populations relying heavily on unprocessed maize as a dietary staple. In traditional Mesoamerican societies, the process of nixtamalization, which involves cooking maize in an alkaline lime solution, was developed to enhance niacin bioavailability and improve overall nutrient digestibility ( Hassan *et al.*, 2023a). This cultural innovation addressed the pellagra problem and enhanced calcium intake through lime absorption, demonstrating a profound synergy between traditional knowledge and nutritional science. Thiamine and riboflavin have both been reported to be less affected by processing, but partially lost during degerming and milling procedures that remove the bran and germ ( Padonou *et al.*, 2023). These vitamins serve important enzymatic roles, such as thiamine as a cofactor in oxidative decarboxylation and riboflavin in electron transport chains. Furthermore, folate, which is an essential component for nucleotide biosynthesis and fetal neural development, is moderately concentrated in maize, typically ranging from 40 to 70 µg per 100 grams of dry kernel ( Xiao *et al.*, 2022). Although folate losses can occur during storage and cooking, breeding efforts are currently underway to enhance folate content through genomic approaches. In areas where maize constitutes a major dietary staple, the contribution of these B-complex vitamins, though often underestimated, is significant for public health, particularly among pregnant women and children ( Glatzel *et al.*, 2025).

### Essential minerals and bioavailability constraints

Mineral content in maize is characterized by moderate levels of iron, zinc, magnesium, phosphorus, and calcium, yet their nutritional contribution is often constrained by bioavailability challenges. A key limiting factor is phytic acid, a natural anti-nutrient that chelates divalent cations such as Fe²⁺ and Zn²⁺, forming insoluble complexes in the gastrointestinal tract that hinder absorption ( Sharma *et al.*, 2022b). This biochemical interaction is particularly problematic in populations where maize is consumed as a staple and access to mineral-rich animal products is limited, thereby exacerbating risks of iron-deficiency anemia and zinc-related growth impairments ( Nsabimana *et al.*, 2024a).

Iron and zinc concentrations in maize grain typically range from 15 to 35 mg/kg, but their poor bioavailability has prompted significant research into genetic and agronomic interventions. Plant breeders have developed low phytate (lpa) maize varieties that reduce phytate content, thereby improving mineral absorption. In parallel, bio-fortified genotypes with intrinsically higher Fe and Zn levels have been released through collaborative programs such as HarvestPlus ( Bouis & Saltzman, 2017). Agronomic bio-fortification, involving soil application or foliar sprays of zinc and iron fertilizers, has also proven effective in enhancing grain mineral concentrations, complementing genetic approaches ( Cakmak, 2008; Prasanna *et al.*, 2020b). Recent trials in South Asia and Central America demonstrated that zinc bio-fortified maize significantly improved serum zinc levels in children, underscoring its potential as a large-scale nutritional intervention ( Gupta *et al.*, 2025a). These findings highlight the dual importance of genetic and agronomic bio-fortification in addressing micronutrient deficiencies through staple crops.

Beyond Fe and Zn, maize also provides other essential minerals. Magnesium, present at concentrations of 100–120 mg per 100 g, is more readily absorbed compared to iron and zinc. It plays a critical role in over 300 enzymatic reactions, including ATP metabolism, neuromuscular transmission, and DNA repair. Magnesium insufficiency can become clinically relevant in maize-based diets that lack complementary sources such as legumes or leafy vegetables. Phosphorus is abundant in maize, ranging from 250 to 400 mg per 100 g, but much of it is stored as phytate phosphorus, which remains inaccessible to monogastric organisms. This further reinforces the importance of low phytate breeding strategies to improve mineral utilization. Calcium content, on the other hand, is naturally low in maize, limiting its contribution to bone mineralization and muscular function ( Aggarwal *et al.*, 2022). Traditional processing techniques such as nixtamalization, however, substantially enhance calcium content by incorporating lime during cooking, sometimes increasing levels up to tenfold depending on lime concentration. Nixtamalization also improves niacin release, making it a critical cultural practice that enhances the micronutrient profile of maize-based diets ( Hassan *et al.*, 2023b).

These findings illustrate that while maize provides a baseline of essential minerals, its nutritional impact is shaped by both intrinsic limitations and external interventions. Genetic innovations such as low phytate and bio-fortified varieties, agronomic bio-fortification strategies, and traditional processing methods all contribute to improving mineral bioavailability and public health outcomes. Table 2 consolidates these insights by summarizing the biochemical roles, estimated content ranges, bio-availability considerations, and public health relevance of key minerals in maize. This integrated framework provides a practical lens for understanding how maize-based diets can either exacerbate or mitigate micronutrient deficiencies in vulnerable populations, thereby guiding agricultural, nutritional, and policy strategies.

**Table 2.** Summary of the Micronutrient Profile of *Zea mays L.*

Micronutrient	Content Range	Functional Role	Bioavailability Considerations	Public Health Impact	References
β-Carotene	1–15 µg/g (biofortified)	Precursor of retinol (vision, immunity, cell differentiation)	Enhanced in orange maize; limited in yellow maize	Prevents vitamin A deficiency; supports maternal/child health	( Amanjyoti *et al.*, 2024).
γ-Tocopherol (Vitamin E)	100–180 mg/kg (germ/oil)	Antioxidant, immune modulation	Stable in high-oil hybrids; sensitive to oxidation	Reduces oxidative stress and CVD risk	( Beer *et al.*, 2024).
Niacin (Vitamin B3)	1–2 mg/100 g (native)	Redox coenzyme (NAD/ NADP), DNA repair	Bound as niacytin; improved via nixtamalization	Pellagra prevention	( Glatzel *et al.*, 2025).
Thiamine (B1)	~0.3 mg/100 g	Carbohydrate metabolism	Lost during milling	Supports energy metabolism	( Padonou *et al.*, 2023).
Riboflavin (B2)	~0.1 mg/100 g	Electron transport (FAD, FMN)	Milling and oxidation losses	Essential for growth, neural health	( Malik *et al.*, 2023).
Folate (B9)	40–70 µg/100 g	DNA synthesis, fetal neural development	Heat-labile; biofortification under exploration	Prevents neural tube defects	( Arlappa *et al.*, 2024)
Iron	2–3 mg/100 g	Hemoglobin synthesis	Poor absorption due to phytate	Biofortified maize under development	( Nsabimana *et al.*, 2024a).
Zinc	1.5–2.5 mg/100 g	Enzyme cofactor, immune defense	Enhanced via biofortification	Reduces stunting and infection risk	( Afolami *et al.*, 2021).
Magnesium	100–120 mg/100 g	Cofactor in >300 enzymes	Moderately bioavailable	Supports metabolic homeostasis	( Hassan *et al.*, 2023a).
Calcium	5–20 mg/100 g (native)	Bone mineralization, neuromuscular signaling	Greatly enhanced via nixtamalization	Contributes to skeletal health	( Sharma *et al.*, 2022a).

**Legend:** *This table summarizes the levels of essential vitamins and minerals in maize, such as B vitamins, iron, zinc, and magnesium, emphasizing their roles in human nutrition.*

### Macronutrient-micronutrient interactions in *Zea mays* L.

Interactions between macronutrients and micronutrients in maize are increasingly recognized as critical determinants of nutritional outcomes, particularly in populations where maize is a dietary staple. Protein quality, for example, not only influences amino acid availability but also affects mineral utilization. Conventional maize proteins, dominated by zeins, are deficient in lysine and tryptophan, limiting their biological value. The development of Quality Protein Maize (QPM) has addressed this limitation by increasing non-zein proteins, thereby enhancing nitrogen retention and improving overall protein digestibility ( Joshi *et al.*, 2022; Prasanna *et al.*, 2001). Improved protein quality has downstream effects on micronutrient metabolism, as adequate amino acid intake supports hemoglobin synthesis and enzymatic functions that depend on iron and zinc cofactors.

Carbohydrate fractions also play a significant role in shaping mineral bioavailability. Resistant starch and dietary fiber can bind minerals in the gastrointestinal tract, reducing their absorption. More importantly, phytate phosphorus, which constitutes the majority of phosphorus in maize, strongly chelates iron and zinc, forming insoluble complexes that limit their uptake ( Sharma *et al.*, 2022b). This interaction explains why iron and zinc deficiencies are common in maize-dependent populations, despite moderate grain concentrations of these minerals ( Nsabimana *et al.*, 2024b). Breeding efforts to develop low-phytate (lpa) maize genotypes have shown promise in mitigating this challenge, as reduced phytate levels enhance the bioavailability of both macronutrients and micronutrients. Lipids in maize germ oil provide another important interaction pathway. Rich in unsaturated fatty acids, maize oil facilitates the absorption of fat-soluble vitamins such as A, E, and K. This relationship is particularly relevant in the context of provitamin A biofortified maize (orange maize), where carotenoid bioavailability is highly dependent on adequate dietary fat intake ( Pixley *et al.*, 2019). Thus, lipid fractions not only contribute to energy density but also act as carriers that enhance micronutrient utilization, linking macronutrient composition directly to vitamin status.

Agronomic biofortification further illustrates the importance of these interactions. Soil and foliar application of zinc and iron fertilizers have been shown to increase grain mineral concentrations, but their effectiveness is maximized when antinutrient levels are reduced ( Cakmak, 2008; Prasanna *et al.*, 2020b). Recent intervention trials demonstrated that zinc-biofortified maize improved serum zinc levels in children, highlighting how mineral enrichment strategies must account for macronutrient–micronutrient dynamics to achieve meaningful health outcomes ( Gupta *et al.*, 2025b). Collectively, these studies demonstrate that maize’s nutritional impact cannot be understood in isolation of macronutrient–micronutrient interactions. Protein quality influences mineral metabolism, carbohydrate fractions modulate mineral absorption, and lipid fractions facilitate vitamin uptake. Biofortification strategies whether genetic, agronomic, or processing-based must therefore adopt an integrated perspective that considers these interactions. Such an approach ensures that improvements in one nutrient domain do not inadvertently compromise another, and that maize-based interventions deliver holistic benefits to vulnerable populations.

## Bioactive phytochemicals in maize

One of the most versatile crops grown in a variety of agroclimatic zones is maize ( Singh *et al.*, 2021). Another name for it is "the poor man's nutri-cereal." About 4.5 billion people in 94 developing countries rely on it as a food crop, providing around 30% of their daily caloric needs. 63% of the world's maize crop is used as animal feed, and it is also a major source of oil, starch, biofuel, etc ( Azadi *et al.*, 2022). The majority of maize genotypes are said to have roughly 67–72% starch, 8% protein, 2–3% fibre, 12–15% moisture, 2–4% fat, and 1.5% minerals ( Azadi *et al.*, 2022). The leaves, kernels, and maize silk have been reported to contain phytochemical secondary metabolites such as saponin, allantoin, sterol, stigmasterol, alkaloids, hordenine, and polyphenols.

Plant metabolism, either primary or secondary, produces phytochemicals. They fight against rivals, diseases, and predators and are vital to plant growth ( Sánchez-Nuño *et al.*, 2024). A variety of phytochemicals are found in maize. Some of these phytochemicals function as antioxidants, neutralising free radicals and removing their potential for damage. Because of their potential as antioxidants, carotenoids and flavonoids are the phytochemicals that have been investigated the most. They strengthen the immune system, lower the risk of some malignancies, and support bone, cardiovascular, visual, and brain health ( Swati *et al.*, 2024). The main factors responsible for the health benefits of maize phytochemicals are their high antioxidant content, antiradical activity, anti-mutagenesis, anti-carcinogenesis, anti-inflammatory, and enzyme inhibitory activity. Regretfully, when the product is ground, processed, and stored, the majority of the phytochemicals are broken down and destroyed ( Swati *et al.*, 2024).

Phenolic acid, which is typically present in whole grains, is the most basic and prevalent phenolic chemical. Coumaric, caffeic, protocatechuic, hydroxybenzoic, vanillic, syringic, sinapic, ferulic, and gallic acids are the most common phenolic acids in maize ( Zhu *et al.*, 2024). Ferulic acid is the primary ingredient that makes up 70% of the total phenolic acids in maize, which averages about 255 mg/100 g ( Swati *et al.*, 2024). Studies conducted both *in vitro* and *in vivo* demonstrate the intricacy of ferulic acid's antioxidant mode of action, which is primarily based on the neutralisation of free radicals and the suppression of the production of reactive oxygen species (ROS) or nitrogen. Furthermore, this acid is in charge of chelating protonated metal ions like Cu(II) and Fe(II) ( Ye *et al.*, 2022). Ferulic acid increases the activity of scavenger enzymes and inhibits the enzymes that catalyse the production of free radicals in addition to being a free radical scavenger. Its chemical structure has a direct connection to it. Additionally, it has demonstrated lipid peroxidation-inhibiting action ( Li *et al.*, 2022). Low in toxicity, ferulic acid offers a wide range of physiological properties, such as anti-inflammatory, antibacterial, anticancer (such as skin, breast, colon, and lung cancer), antiarrhythmic, and antithrombotic effects. Additionally, it showed immunostimulant and anti-diabetic benefits, decreased damage to nerve cells, and might aid in cell repair, as illustrated in Table 3 ( Zduńska *et al.*, 2018).

**Table 3.** Summary of Major Phytochemicals in Maize and Their Health Implications.

Phytochemical Class	Major Compounds	Biological Functions	Health Implications
Phenolics	Ferulic acid, Gallic acid, Caffeic acid, Coumaric acid, Sinapic acid, Vanillic acid	Antioxidant, free radical scavenging, metal ion chelation, enzyme modulation, angiogenesis regulation	Anti-inflammatory, anticancer (skin, breast, colon, lung), antidiabetic, neuroprotective, cell repair
Flavonoids	Quercetin, Kaempferol, Apigenin (general flavonoids in maize silk and kernels)	Antioxidant, anti-radical, anti- glycation, oxidative stress modulation	Cardioprotective, hepatoprotective, anti-diabetic, anti-obesity, anticancer, anti-fatigue
Carotenoids	β-Carotene, α-Carotene, Zeaxanthin, Lutein, β- Cryptoxanthin	Provitamin A activity, antioxidant, visual health, photosynthesis support, pollinator attraction	Prevention of vitamin A deficiency, improved vision, reduced child mortality, and immune and skin health
General Antioxidants	A combination of phenolics, flavonoids, carotenoids, vitamins, and minerals	Free radical neutralization, oxidative damage prevention, anti- mutagenesis, anti-inflammatory, and enzyme inhibition	Reduced risk of cardiovascular diseases, diabetes, obesity, and digestive disorders

**Legend:** *This table details the presence of bioactive compounds in maize, including phenolics, flavonoids, and carotenoids, and their associated antioxidant properties.*

By altering the activity of the key players, vascular endothelial growth factor (VEGF), platelet-derived growth factor (PDGF), and hypoxia-inducible factor 1 (HIF-1), ferulic acid has been demonstrated to have an angiogenesis effect ( Zduńska *et al.*, 2018). While gallic acid inhibits the formation of new blood vessels in ovarian cancer cells, ferulic acid has been demonstrated in studies involving human umbilical vein endothelial cells to increase the expression of VEGF and PDGF and the quantity of hypoxia-induced HIF-1, which produces responses to hypoxia ( He *et al.*, 2016; Sánchez-Nuño *et al.*, 2024).

Because of their strong antioxidant qualities, flavonoids can scavenge free radicals and stop protein glycation ( Meena *et al.*, 2022). They have also been found to have anti-oxidant and anti-radical qualities, which are connected to a number of preventative qualities, including cardioprotective, hepatoprotective, anti-inflammatory, anti-obesity, anti-diabetic, and anti-cancerous ( Meena *et al.*, 2022). Bioactive phytochemicals such as polysaccharides, vitamins, terpenoids, steroids, alkaloids, tannins, saponins, volatile oils, and sugars are abundant in maize silk. Research has demonstrated that the flavonoids found in maize silk have a protective effect against oxidative stress and have the ability to prevent weariness in mice ( Swati *et al.*, 2024). Every component of the maize plant contains beneficial phytochemical substances ( Meena *et al.*, 2022). When these phytochemicals are extracted from biomass, the biomass's qualities and potential for biological conversion to energy sources like biogas or bioethanol are improved ( Oleszek *et al.*, 2019).

On the other hand, carotenoids are the cause of the different colouring pigments, such as red, orange, and yellow. Because they are antioxidants and a precursor to vitamin A, carotenoids are advantageous ( Suriano *et al.*, 2021). A lack of vitamin A in humans can result in night blindness, increased mortality and stunted growth in children, dry skin, and infertility in those living in underdeveloped nations ( Natesan *et al.*, 2020). The various processing factors, like homogenization and heat treatments, may enhance carotenoid bioavailability ( Song *et al.*, 2021). In addition to serving as precursors of vitamin A, carotenoids play a number of vital functions, including attracting pollination-causing insects, preventing photooxidation, and facilitating vital pigment photosynthetic processes. The carotenoid concentration of maize is divided into two categories: (i) xanthophylls (lutein, zeaxanthin, and β-cryptoxanthin, which only include an oxygen group) and (ii) carotenes (α-carotene and β-carotene, which have carbon and hydrogen) as shown in Table 3. During human metabolic activity, the β-carotene isoform, which has the highest provitamin A activity known, readily transforms into vitamin A ( Sheng *et al.*, 2018).

Fibre, vitamins, minerals, and phytochemicals are among the many nutrients and bioactive substances found in whole grain maize. A growing body of research indicates that eating whole grains regularly lowers the chance of acquiring chronic illnesses like cardiovascular disease, type II diabetes, overweight and obesity, and digestive issues ( Sánchez-Velázquez *et al.*, 2023).

## Effect of genotype on the influence of variety and soil conditions

The genotype of *Zea mays* L. plays a pivotal role in determining its nutritional composition, influencing both proximate and micronutrient values. Different maize varieties exhibit diverse levels of protein, fat, carbohydrates, and essential minerals, which directly affect their nutritional quality and applicability in various diets. For instance, Kabir *et al.* (2019) reported that protein content among five maize varieties ranged from 13.11% in BHM-15 to 10.96% in BHM-8, while fat content varied between 5.44% in BHM-15 and as low as 0.19% in BHM-8. Carbohydrate content was also variable, with BHM-13 showing the highest value at 82.40%, whereas BHM-5 recorded the highest fiber content at 2.07%. These findings highlight the genetic diversity within maize germplasm and its implications for dietary energy and macronutrient balance.

Micronutrient concentrations also vary significantly across genotypes. Thakur *et al*. (2015) observed kernel iron levels ranging between 22.59 and 41.03 mg/kg, while zinc concentrations varied from 19.38 to 32.59 mg/kg. Carotenoid content likewise differed among genotypes, with values ranging from 10.72 to 27.61 µg/g, and CML162 exhibiting the highest carotenoid level. Such variability underscores the potential of targeted breeding programs to enhance micronutrient density in maize. Beyond grain quality, forage traits are also genotype-dependent. Hybrid maize genotypes generally demonstrate superior feed value due to higher dry matter content and lower fiber, resulting in improved digestibility. The Amarillo variety, for example, exhibited high stability and reduced silage loss, making it favorable for forage production ( Alvarado-Ramirez *et al.*, 2023).

Recent advances in biofortification have further emphasized the role of genotype in nutrient accumulation. Quality Protein Maize (QPM) hybrids such as Obatanpa, Shakti, and HQPM series combine improved amino acid profiles with enhanced mineral uptake efficiency, offering dual benefits in macronutrient and micronutrient quality ( Joshi *et al.*, 2022; Prasanna *et al.*, 2001). Similarly, DHM 117, a medium-duration hybrid tested in India, has shown responsiveness to zinc and iron fertilization, with agronomic biofortification trials reporting significant increases in grain mineral concentrations under optimized soil fertility management ( Urrea-Benítez, 2024). Foliar application of 0.5–1.0% ZnSO _4_ at tasseling and grain-filling stages has been shown to increase grain zinc concentrations by 20–40%, while basal soil applications of 25–50 kg/ha ZnSO _4_ or FeSO _4_ improve mineral content depending on soil type and cultivar ( Xue *et al.*, 2023). Importantly, feeding trials with zinc-biofortified maize demonstrated improved serum zinc levels in children, confirming that agronomic interventions translate into tangible nutritional benefits ( Gupta *et al.*, 2025b). Soil conditions further modulate genotype performance. In calcareous soils, iron solubility is reduced, while acidic soils often enhance Fe uptake but limit Zn availability. Cropping systems also influence mineral dynamics; maize grown after legumes benefits from improved soil micronutrient status due to biological nitrogen fixation and root exudates that mobilize Zn and Fe ( Cakmak, 2008). These findings highlight the importance of integrating genotype selection with site-specific agronomic practices to maximize nutrient density and bioavailability.

Collectively, these studies demonstrate that maize’s nutritional quality is shaped by the interplay of genotype, variety, and soil conditions. Incorporating promising cultivars into agronomic biofortification programs, alongside dosage optimization and bioavailability studies, provides a comprehensive framework for improving grain nutrient density. This integrated approach ensures that maize biofortification strategies are both scientifically robust and nutritionally impactful, addressing micronutrient deficiencies in vulnerable populations.

## Agronomic practices and environment on the influence of variety and soil conditions

Various agronomic strategies significantly influence the nutritional profile of *Zea mays L.*, including fertilization, weed management, nitrogen regulation, and varietal selection. These practices do not only boost the macro- and micronutrient contents of the grain, they also contribute to improved yield and overall crop quality. Fertilization, whether organic, mineral, or combined, has been shown to affect maize nutrition positively. For example, mixed fertilization significantly increased protein and mineral contents compared to unfertilized plots ( Marsaro Júnior *et al.*, 2007), while the application of tobacco waste compost and poultry manure improved grain yield and boosted levels of nitrogen, phosphorus, and potassium ( Cercioglu, 2023). Weed management strategies such as the use of wood shavings as mulch were found to improve nutrient uptake and grain quality while effectively suppressing weed growth ( Omovbude *et al.*, 2017). Proper nitrogen management is equally critical, as timely nitrogen application enhances plant health, kernel development, and ear filling, resulting in improved grain yield and quality ( Kafle *et al.*, 2023; Szulc *et al.*, 2023). However, excessive reliance on chemical fertilizers may pose environmental risks, such as nutrient leaching and soil degradation, highlighting the need for balanced and sustainable fertilizer use.

In addition to agronomic practices, the maize variety also plays a vital role in determining nutrient content. Creole varieties generally exhibit higher concentrations of phosphorus and potassium compared to high-yielding hybrids ( Ferreira *et al.*, 2014), while nutrient composition, including proteins and lipids, varies significantly among cultivars. Some hybrids are noted for maintaining consistent nutrient distribution across varying environments ( Dragičević *et al.*, 2014). Furthermore, soil type and moisture availability also affect nutrient levels; maize cultivated on fertile soils tends to have higher nutrient concentrations, while drought conditions may reduce starch but increase protein levels ( Dragičevic *et al.*, 2014). While genotype and soil are major determinants of nutritional outcomes, other dynamic factors such as pest resistance and market demand also shape agronomic decisions. The complexity of these interactions underscores the importance of integrated, environmentally conscious agricultural practices to optimize both yield and nutritional value in maize production.

## Implications for nutrition and human health

Micronutrient deficiencies, particularly in vitamin A, iron, zinc, and folate, remain major global public health concerns, especially in low- and middle-income countries where cereal-based diets dominate ( Bailey *et al.*, 2015; Gupta *et al.*, 2020; Olalekan, 2024; Tulchinsky, 2010). These deficiencies, often termed “hidden hunger,” impair immune function, hinder growth and cognitive development, and increase the risk of maternal and child mortality ( Das & Padhani, 2022). As a widely consumed staple crop, *Zea mays L.* (maize) presents a strategic opportunity for addressing these nutritional gaps through biofortification, agronomic enhancement, and dietary diversification ( Maqbool *et al.*, 2018; Prasanna *et al.*, 2020a). Beyond its caloric value, maize is increasingly recognized for its potential to alleviate micronutrient deficiencies, making it a vital tool in public health nutrition ( Goredema-Matongera *et al.*, 2021a).

### Vitamin A

Vitamin A Deficiency (VAD) is a leading cause of preventable blindness and mortality among children and pregnant women. Conventional white maize contains negligible provitamin A carotenoids; however, biofortified yellow and orange maize enriched with β-carotene has demonstrated significant potential in reducing VAD ( Akhtar *et al.*, 2013; Baytekus *et al.*, 2019; Maqbool *et al.*, 2018). Initiatives like HarvestPlus have facilitated the introduction of these varieties across several African countries, with clinical studies showing improved serum retinol levels and decreased VAD risk ( Shwetha *et al.*, 2020). Additionally, carotenoids in biofortified maize remain relatively stable during processing, enhancing their practical dietary impact.

### Iron

Iron plays a vital role in oxygen transport, DNA synthesis, and energy metabolism, making it indispensable for human health across all life stages. Iron deficiency remains the most prevalent cause of nutritional anemia worldwide, negatively affecting cognitive development, immune function, and physical productivity, particularly among women and children ( Coad & Pedley, 2014; Madhan *et al.*, 2025). Infants and young children have proportionally higher iron needs relative to body weight, requiring approximately **0.3–0.5 mg/kg/day**, while adults average closer to **0.1 mg/kg/day**. In absolute terms, recommended dietary allowances (RDAs) specify that children aged 1–3 years need about **7 mg/day**, adolescent boys require **11 mg/day**, and adolescent girls up to **15 mg/day** due to menstrual losses. Adult men require **8 mg/day**, whereas women of reproductive age need **18 mg/day**, with requirements rising to **27 mg/day during pregnancy to** support fetal growth and increased blood volume ( FAO/WHO, 2001; Health Canada, 2023; NIH, 2023a).

While maize is a staple food in many regions, it naturally contains limited bioavailable iron. This limitation is compounded by the presence of phytic acid, which chelates iron and reduces absorption. To address this challenge, selective breeding has led to the development of iron dense cultivars with enhanced grain mineral concentrations ( Goredema-Matongera *et al.*, 2021b). In addition, strategies such as the incorporation of low phytate maize genotypes and traditional processing methods—including fermentation and nixtamalization—have been shown to improve iron bioavailability by reducing antinutrient content and increasing mineral solubility ( Nsabimana *et al.*, 2024a). These approaches, when combined with agronomic biofortification practices, provide a comprehensive framework for addressing iron deficiency in maize dependent populations.

### Zinc requirements and nutritional implications

Zinc is essential for immune function, growth, and cellular repair, serving as a cofactor for over 300 enzymes involved in DNA synthesis, protein metabolism, and wound healing. Its deficiency is strongly associated with stunted growth, increased susceptibility to infections, and delayed wound healing in children, making it a critical micronutrient for early development and immune resilience ( Krebs *et al.*, 2014). Infants require approximately **0.3 mg/kg/day**, translating to about **2 mg/day** for those aged 0–6 months, while children aged 1–3 years need **3 mg/day**. Adolescent boys require **11 mg/day** and girls **8–9 mg/day**, reflecting differences in growth and metabolic demand. Adult men require **11 mg/day**, and adult women need **8 mg/day**, with requirements increasing to **11–12 mg/day during pregnancy** and **12 mg/day during lactation** to support fetal and infant development ( FAO/WHO, 2001; Health Canada, 2023; NIH, 2023b).

In maize-dependent populations, zinc deficiency is exacerbated by the presence of phytic acid, which binds zinc and reduces its absorption. Strategies to overcome this limitation include the development of **zinc-biofortified maize varieties**, which have shown promise in improving dietary intake and absorption of zinc, particularly when consumed in diverse diets ( Chomba *et al.*, 2015; Maqbool & Beshir, 2019). As with iron, reducing phytic acid content through low-phytate maize genotypes or traditional processing methods such as fermentation and nixtamalization plays a pivotal role in enhancing zinc uptake ( Sarkhel & Roy, 2022). These approaches, combined with agronomic biofortification practices such as foliar zinc application, provide a comprehensive framework for addressing zinc deficiency in vulnerable populations.

### Folate

Folate is crucial for cell division, DNA synthesis, and amino acid metabolism, especially during periods of rapid growth such as pregnancy and infancy ( Paul *et al.*, 2025; Sobral *et al.*, 2024). Folate deficiency during early pregnancy is linked to neural tube defects and other congenital abnormalities ( Kancherla, 2023). Although maize is not inherently rich in folate, genotypes such as sweet corn and certain inbred lines By855, Si273, GY386B, and CML118, have been identified to contain moderate folate and other B vitamin levels ( Safiul Azam *et al.*, 2022; Xiao *et al.*, 2022). These serve as promising germplasm for biofortification. Incorporating maize into diversified diets can help improve folate intake, particularly in regions with limited access to leafy greens and legumes.

### Protein and bioactive compounds

Maize’s nutritional value extends beyond micronutrients. Quality Protein Maize (QPM), bred to enhance lysine and tryptophan content amino acids limited in conventional maize, offers improved protein quality for populations with limited access to animal protein ( Hossain *et al.*, 2019; Jilo, 2022). Moreover, pigmented maize varieties contain abundant flavonoids, polyphenols, and phytosterols that exhibit antioxidant, anti-inflammatory, and potential anticancer properties ( Sánchez-Nuño *et al.*, 2024). These bioactive compounds further enhance maize’s role as a functional food.

### Broader nutritional impact

In addition to specific nutrients, maize contributes dietary fiber, essential fatty acids, and minerals that support gut health, metabolic regulation, and chronic disease prevention ( Blandino *et al.*, 2017; Ekpa *et al.*, 2018; Meena *et al.*, 2022). Its adaptability to different climates, affordability, and cultural acceptability position maize as a practical, scalable solution for advancing nutritional equity. Through integrated approaches like biofortification, agronomic innovation, and dietary diversification, maize stands as a cornerstone crop in the global effort to reduce malnutrition and promote human health.

## Conclusion

Maize ( *Zea mays L.*) is one of the foundations of the world's food security and a valuable source of macronutrients - carbs, protein, and lipids, important micronutrients, and health-promoting biologically active compounds. Biofortified crops like Quality Protein Maize (QPM) and provitamin A varieties have acquired crucial innovations in terms of gradually mitigating protein-energy malnutrition and micronutrient deficiencies, mainly in low- and middle-income areas. More so, phytochemical contents of maize, such as ferulic acid, flavonoids, and carotenoids, offer potential antioxidant, anti-inflammatory, and anticarcinogenic effects that make maize more of an anticarcinogenic food than the actual staple crop it is. However, some challenges face the nutritional potential of maize; factors that limit the nutritional potential of maize include low bioavailability of minerals in the presence of phytates, an extensive knowledge base to be developed in becoming an essential element in maize breeding, and underutilisation of maize by-products, despite its vast nutritional potential. It is necessary to intensify interdisciplinary research and policy initiatives to fully realise the potential of maize in sustainable food systems in the context of public health nutrition.

## Future directions

Although the nutritional improvement of maize has progressed to a high level, it still has various important gaps and research areas in front. The metabolomic profiling of maize genotypes under stress conditions due to climate change is one of the most appealing unexplored areas. The quality of crops is now threatened by climate change, and the combination of metabolomics and transcriptomics may demonstrate the influence of stress response on scenarios that involve nutrient biosynthesis mechanisms ( Swati *et al.*, 2024). The relevant study of these biochemical modifications will become key in the development of resilient maize cultivars that still retain or rather enhance the nutritional content even under environmental stress.

The second aspect that should be addressed is that of bioavailability of important microelements, in particular, iron, zinc, and niacin. Although biofortified maize varieties have elevated the consumption of nutrients, they are compromised by the presence of antinutritional compounds that include phytates and bound forms of nutrients such as niacytin ( Hrubša *et al.*, 2022; Sharma *et al.*, 2022a). Considerable urgency now exists to conduct additional human trials and clinical studies on the absorption of utilization of these nutrients after intake, including following different processing and cooking conditions ( Nsabimana *et al.*, 2024a). To this end, greater levels of mineral absorption may be had by the development of low-phytate or enzyme-enhanced maize.

Finally, emerging genetic technologies such as CRISPR-Cas9 hold immense potential for precision breeding of maize with enhanced nutritional traits. Unlike traditional breeding or transgenic approaches, CRISPR allows targeted editing of genes responsible for amino acid synthesis, phytate reduction, or carotenoid accumulation without foreign DNA insertion, addressing both regulatory and consumer acceptance concerns ( Li *et al.*, 2022). For example, knocking out the IPK1 gene has shown promise in reducing phytic acid content in cereals, improving mineral bioavailability. Further integration of genomic selection, gene editing, and omics-driven phenotyping is essential for developing next-generation maize cultivars with optimized nutritional and agronomic traits. In conclusion, future research must focus on bridging the gap between nutrient content and bioefficacy, harnessing cutting-edge technologies for targeted enhancement, and aligning agricultural innovation with climate resilience and human health goals.

## Ethics and consent statement

Ethical approval and consent were not required.

## Data Availability

No data associated with this article
