# Supplementary material for: Nutritional composition of Zea mays L: A comprehensive Review of Macronutrients, Micronutrients, and Bioactive Compounds
Source: Open Res Afr. 2026 Feb 13;8:15. Originally published 2025 Sep 10. [Version 2] doi: 10.12688/openresafrica.16056.2 (PMC13000404; doi:10.12688/openresafrica.16056.2)
Supplement: Peer review history

# Reviewer report: Reviewer response for version 2

**DOI:** 10.21956/openresafrica.17556.r33932

**Published:** 2026-03-18

**Authors:** Pradyuman Kumar

**Affiliations:** 1 Department of Food Engineering and Technology, Sant Longowal Institute of Engineering and Technology, Longowal, Punjab, India

1. In Table No. 1, the serial number is not mentioned properly.
2. In Table no.2 and Table no 3, there is no serial number. Keep the same formatting.
3. The biofortification part can also be added for Zea mays L.
4. A paragraph for the application part of the maize can also be added.
5. In the abstract part, shorten long sentences for clarity.
6. In the conclusion, clearly mention what future research should focus on.
7. Clarify the statement “maize remains the firstly produced cereal in the world” with FAO statistics, in the introduction, and provide more recent production data (post-2021) to strengthen relevance.
8. In the carbohydrate section, add a short comparison of how easily maize starch is digested compared to starch from other cereals like wheat and rice.
9. In the protein section, add more explanation about how Quality Protein Maize (QPM) helps the body use nitrogen better.
10. In the micronutrient section, add a clear explanation of how the bioavailability of nutrients in maize can be improved.
11. Add quantitative data for carotenoids, flavonoids, and phenolic acids in maize.
12. Compare these phytochemical levels in different maize types for example, yellow maize, white maize, and biofortified varieties. This will help readers see how nutrient content changes across varieties and why biofortified maize is more beneficial.
13. Create a summary table for vitamins, minerals, and phytochemicals, just like the one already included for macronutrients. This will make the paper easier to understand and give readers a quick visual overview of the key information.
14. Break long sentences into shorter ones so the text is easier to read.
15. Use the same style for all references (author name, year, format).
16. Do not repeat the same citation unless it adds new information.
17. In the conclusion, clearly connect maize’s nutrition benefits to real-world policies, such as school feeding programs and food fortification projects.
18. Add a short section on future research needs. For example, mention genetic engineering and agronomic biofortification as ways to improve nutrient content and reduce antinutritional factors. This will make the paper more practical and show how research can guide both policy and innovation.

Is the review written in accessible language?

Partly

Are all factual statements correct and adequately supported by citations?

Partly

Are the conclusions drawn appropriate in the context of the current research literature?

Yes

Is the topic of the review discussed comprehensively in the context of the current literature?

Yes

Reviewer Expertise:

New product development, bioactive compounds, food macromolecules and micro molecules

I confirm that I have read this submission and believe that I have an appropriate level of expertise to confirm that it is of an acceptable scientific standard, however I have significant reservations, as outlined above.

# Reviewer report: Reviewer response for version 2

**DOI:** 10.21956/openresafrica.17556.r33750

**Published:** 2026-02-16

**Authors:** Augustine Augustine

**Affiliations:** 1 Karunya Institute of Technology and Sciences,, Coimbatore, Tamil Nadu, India

Corrections noted and appropriate.

Is the review written in accessible language?

Yes

Are all factual statements correct and adequately supported by citations?

Yes

Are the conclusions drawn appropriate in the context of the current research literature?

Yes

Is the topic of the review discussed comprehensively in the context of the current literature?

Yes

Reviewer Expertise:

Agronomic biofortification, nutrient sensing, INM, and digital platforms for maize biofortification and value chain enhancement, maize cropping systems

I confirm that I have read this submission and believe that I have an appropriate level of expertise to confirm that it is of an acceptable scientific standard.

# Response

**Published:** 2026-02-19

**Authors:** Michael Okon

**Affiliations:** Biochemistry, Kampala International University - Western Campus, Bushenyi, Western Region, Uganda

Thank you so much Sir for your keen review. After attending to all your points, your thoroughness has helped me improve scientifically.

 Regards!

# Reviewer report: Reviewer response for version 1

**DOI:** 10.21956/openresafrica.17231.r32862

**Published:** 2025-11-25

**Authors:** Augustine Augustine

**Affiliations:** 1 Karunya Institute of Technology and Sciences,, Coimbatore, Tamil Nadu, India

The introduction is well written, mentioning the problems and solutions and the focus precisely.

 Macronutrient : Well defined and clear. Also biofortification related areas can also be covered when QPM is mentioned.

 Micronutrients: Special emphasis can be given to Fe and Zn under the essential minerals. Numerous studies have been conducted on the impact of agronomic biofortification, also known as mineral biofortification, using zinc and iron, which can be included. 

 Interaction studies comparing macronutrients with micronutrients can be included.

 Effect of genotype on the influence of variety and soil conditions: Incorporate some promising cultivars and conduct application studies under Agronomic practices, including dosage and bioavailability of grain nutrients.

 Implications for nutrition and human health: The required levels of iron, zinc, etc., for humans at different stages, such as children, men, and women, can be specified in terms of body weight/gm.

 Future directions:   Done with more clarity

Is the review written in accessible language?

Yes

Are all factual statements correct and adequately supported by citations?

Yes

Are the conclusions drawn appropriate in the context of the current research literature?

Yes

Is the topic of the review discussed comprehensively in the context of the current literature?

Yes

Reviewer Expertise:

Agronomic biofortification, nutrient sensing, INM, and digital platforms for maize biofortification and value chain enhancement, maize cropping systems

I confirm that I have read this submission and believe that I have an appropriate level of expertise to confirm that it is of an acceptable scientific standard.

# Response

**Published:** 2025-12-11

**Authors:** Michael Okon

**Affiliations:** Biochemistry, Kampala International University - Western Campus, Bushenyi, Western Region, Uganda

**Comment: **Macronutrient: Well defined and clear. Also, biofortification related areas can also be covered when QPM is mentioned.

**Response:** This has been addressed in the manuscript (page 7)

**Comment: **Micronutrients: Special emphasis can be given to Fe and Zn under the essential minerals. Numerous studies have been conducted on the impact of agronomic biofortification, also known as mineral biofortification, using zinc and iron, which can be included. 

**Response:**

 This has been addressed in the manuscript (Page 12)

**Comment: **Interaction studies comparing macronutrients with micronutrients can be included.

**Response:** This has been addressed in the manuscript (Page 13)

**Comment: **Effect of genotype on the influence of variety and soil conditions: Incorporate some promising cultivars and conduct application studies under Agronomic practices, including dosage and bioavailability of grain nutrients.

**Response:** This has been addressed in the manuscript (Page 17)

**Comment:** Implications for nutrition and human health: The required levels of iron, zinc, etc., for humans at different stages, such as children, men, and women, can be specified in terms of body weight/gm.

**Response:** This has been addressed in the manuscript (Page 20 and 21)

# Reviewer report: Reviewer response for version 1

**DOI:** 10.21956/openresafrica.17231.r32540

**Published:** 2025-10-16

**Authors:** Kristina Kljak

**Affiliations:** 1 University of Zagreb, Zagreb, Croatia

The manuscript is a comprehensive review of maize from the perspective of macronutrient composition and contents of micronutrients and bioactive compounds. The authors also included nutritional potential in humans.

 Although the intention of the authors is noteworthy, the manuscript has some shortcomings, making it unsuitable for acceptance in this form.

 Firstly, the authors focus on QPM maize or popcorn as examples. What about other varieties?

 Authors should be specific in presenting the results:

- *Production may be by a smallholder farmer in a rain-fed situation, especially in the Global South (Egah et al., 2024).* – This is a specific situation; authors should be clear if they are focusing on this specific production or production in general.
- *Maize lipid profile shows a favourable fatty acid composition, such as the polyunsaturated fatty acids (PUFA), monounsaturated fatty acids (MUFA), and saturated fatty acids.* – The authors are mentioning groups of fatty acids without explaining what is favourable in them.
- *Maize oil belongs to the family of fats rich in unsaturated fatty acids, with many of them being omega-3…* - What is the proportion of omega acids in maize oil?

There are capital letters in the sentence that are not needed. Authors need to check the manuscript for these types of mistakes.

 A large number of sentences is not straightforward and very difficult to understand. Examples are:

- *Maize is grown in every agro-ecological zone (Adiaha, 2017), which grants it some varieties in climates and soil types. *– Sentence is unclear.
- *In addition to consuming maize as whole grains, other forms of maize can be processed into cornmeal, tortillas, flatbreads, starches, and breakfast cereals, among other things (Mehta & Dias, 1999; Shah et al., 2016)*. – The sentence is unclear; maize grain can be processed into these foods.
- *As a functional food, maize provides macronutrients along with bioactive compounds like polyphenols, carotenoids, phenolic acids, flavonoids, phytosterols, and lignins (Sheng et al., 2018), which act as antioxidants and provide disease-preventive actions, Likewise the chances of contracting disease like diabetes, cardiovascular problems, obesity, or digestive disorders are degreased by Regular intake of such foods (Baranowska, 2023) *– The sentence is not clear.
- I *n normal varieties, the value of maize oil content is between 3–5 percent, but in some hybrids and biofortified lines, the percentages go up to 7 pieces…* – revise the sentence.
- *Maize oil belongs to the family of fats rich in unsaturated fatty acids, with many of them being omega-3, making it a very useful dietary and industrial ingredient with significant potential to comprise the macronutrient content of the diet, it is a well-known phytochemical component in maize oil, which is the most plentiful member of the maize species.* – The sentence is too long and difficult to understand.

A large part of the information provided in the text is duplicated in Table 1 and Figure 1. Authors should address this issue and adapt them to be addition to the text and not repetition.

 Authors often repeat the text. For example:

- *The nutritional value of maize derives from its functionality and nutritional value in the macronutrient content of its carbohydrates, proteins, and lipids. Starch is the main carbohydrate and is also a dependable source of energy, and dietary Fibre can increase the health values, especially in the lesser-exploited by-products such as corn silk and husk. Proteins, particularly in QPM, provide better balanced amino acids to overcome the nutritional deficiency, and lipids, especially concentrated in germ, provide the heart-friendly fats and phytochemicals. *
- *One of the most versatile crops grown in a variety of agroclimatic zones is maize (Singh et al., 2021). Another name for it is "the poor man's nutri-cereal." About 4.5 billion people in 94 developing countries rely on it as a food crop, providing around 30% of their daily caloric needs. 63% of the world's maize crop is used as animal feed, and it is also a major source of oil, starch, biofuel, etc (Azadi et al., 2022). The majority of maize genotypes are said to have roughly 67–72% starch, 8% protein, 2–3% fibre, 12–15% moisture, 2–4% fat, and 1.5% minerals (Azadi et al., 2022). The leaves, kernels, and maize silk have been reported to contain phytochemical secondary metabolites such as saponin, allantoin, sterol, stigmasterol, alkaloids, hordenine, and polyphenols.*
- *Fibre, vitamins, minerals, and phytochemicals are among the many nutrients and bioactive substances found in whole grain maize.*

Other comments:

- What about lutein and zeaxanthin? Major carotenoids in maize and important for human health but it was not mentioned.
- What about resistant starch?
- *The nutraceutical potential of maize is a modern concern with maize. As a functional food, maize provides macronutrients along with bioactive compounds like polyphenols, carotenoids, phenolic acids, flavonoids, phytosterols, and lignins* – Delete polyphenols since specific polyphenol compounds are mentioned. What about tocols?
- *Generally, the conventional variants of maize, amongst others, include iron, zinc, and vitamin A…* - Maize does not contain vitamin a but provitamin A compounds.
- *The lipids are present as trace elements…* - they are not elements but compounds.
- *These practices not only enhance macro- and micronutrient contents of the grain but also improve overall yield and quality.* – These practices can affect composition and yield, depending on the practice, and whether this effect is going to be positive. Rephrase the sentence. In addition, some of the practices were mentioned in the text (page 4) but they would better fit in this part.

Is the review written in accessible language?

Partly

Are all factual statements correct and adequately supported by citations?

Partly

Are the conclusions drawn appropriate in the context of the current research literature?

Yes

Is the topic of the review discussed comprehensively in the context of the current literature?

Yes

Reviewer Expertise:

Nutritional value of maize grain (carotenoids, phenolics, tocols, carotenoid bioaviability, starch digestibility kinetics, protein fractions, physical properties)

I confirm that I have read this submission and believe that I have an appropriate level of expertise to confirm that it is of an acceptable scientific standard, however I have significant reservations, as outlined above.

# Response

**Published:** 2025-10-28

**Authors:** Michael Okon

**Affiliations:** Biochemistry, Kampala International University - Western Campus, Bushenyi, Western Region, Uganda

**Reviewer Comment 1** Firstly, the authors focus on QPM maize or popcorn as examples. What about other varieties?

**Response:** *The authors did not focus on QPM. However, the studies included from the search engines predominantly studied QPM.*

**Comment 2** *Production may be by a smallholder farmer in a rain-fed situation, especially in the Global South (Egah et al., 2024).* – This is a specific situation; authors should be clear if they are focusing on this specific production or production in general.

**Response:** The production focuses on production in general and this has been updated as follows: Maize is cultivated extensively across all agro-ecological zones, demonstrating its remarkable adaptability to diverse climates and soil types. This ecological flexibility not only supports its widespread cultivation but also enables the development of region-specific maize varieties tailored to local and global environmental conditions (Adiaha, 2017). Maize production is predominantly carried out by smallholder farmers under rain-fed conditions, making it highly sensitive to climatic variability and resource constraints (Egah et al., 2024).

***Comment 3*** *Maize lipid profile shows a favourable fatty acid composition, such as the polyunsaturated fatty acids (PUFA), monounsaturated fatty acids (MUFA), and saturated fatty acids.* – The authors are mentioning groups of fatty acids without explaining what is favourable in them.

**Response:** The complete statement: “Maize lipid profile shows a favourable fatty acid composition, such as the polyunsaturated fatty acids (PUFA), monounsaturated fatty acids (MUFA), and saturated fatty acids. An example of this is popcorn, which contains PUFA and MUFA as important elements that help in the nutritional value of the kernels (Dada et al., 2023). Maize oil is rich in linoleic acid (a PUFA, 50–60%), oleic acid (a MUFA, 25–35%), and smaller amounts of palmitic and stearic acids (saturated fatty acids, 10 – 15%), making it a heart-healthy oil with applications in cooking and food processing (Mandal et al., 2023)” showed that the fatty acids are important nutritional values and contributes to a healthy heart.

**Comments 4:** **This has been updated to: **Maize oil belongs to the family of fats rich in unsaturated fatty acids, with the highest proportion of about 50-60% being omega-6 (Mandal et al., 2023). This makes it a very useful dietary and industrial ingredient with significant potential to comprise the macronutrient content of the diet, it is a well-known phytochemical component in maize oil, which is the most plentiful member of the maize species.

***Comments 5:*** *Maize is grown in every agro-ecological zone (Adiaha, 2017), which grants it some varieties in climates and soil types.*

**Response:** Maize is cultivated extensively across all agro-ecological zones which demonstrates its remarkable adaptability to diverse climates and soil types. This ecological flexibility not only supports its widespread cultivation but also enables the development of region specific maize varieties tailored to local and global environmental conditions (Adiaha, 2017). Maize production is predominantly carried out by smallholder farmers under rain-fed conditions, making it highly sensitive to climatic variability and resource constraints (Egah et al., 2024).

**Comments 6:** *In addition to consuming maize as whole grains, other forms of maize can be processed into cornmeal, tortillas, flatbreads, starches, and breakfast cereals, among other things (Mehta & Dias, 1999; Shah et al., 2016)*. – The sentence is unclear; maize grain can be processed into these foods.

**Response:** This has been updated to: In addition to its consumption as whole grains, maize ( *Zea mays L.*) is widely processed into a variety of food products, which includes cornmeal, tortillas, flatbreads, starches, and breakfast cereals. These products reflect the functional diversity of maize and its central role in both traditional and industrial food systems (Mehta & Dias, 1999; Tajamul et al., 2016).

**Comments 7:** *As a functional food, maize provides macronutrients along with bioactive compounds like polyphenols, carotenoids, phenolic acids, flavonoids, phytosterols, and lignins (Sheng et al., 2018), which act as antioxidants and provide disease-preventive actions, Likewise the chances of contracting disease like diabetes, cardiovascular problems, obesity, or digestive disorders are degreased by Regular intake of such foods (Baranowska, 2023) *– The sentence is not clear.

**Response:** This has been addressed in response 6.

**Comment 8:** I *n normal varieties, the value of maize oil content is between 3–5 percent, but in some hybrids and biofortified lines, the percentages go up to 7 pieces…* – revise the sentence.

**Response:** In conventional maize varieties, the oil content typically ranges between 3–5%, but in some hybrids and biofortified lines, it can reach up to about 7%.

**Comment 9:** *Maize oil belongs to the family of fats rich in unsaturated fatty acids, with many of them being omega-3, making it a very useful dietary and industrial ingredient with significant potential to comprise the macronutrient content of the diet, it is a well-known phytochemical component in maize oil, which is the most plentiful member of the maize species.* – The sentence is too long and difficult to understand.

**Response:** For better understanding, the concise updated sentence reads:

 Maize oil belongs to the family of fats rich in unsaturated fatty acids, with the highest proportion of about 50-60% being omega-6 (Mandal et al., 2023). This makes it a very useful dietary and industrial ingredient with significant potential to comprise the macronutrient content of the diet, it is a well-known phytochemical component in maize oil, which is the most plentiful member of the maize species.

**Comment 10:** *Fibre, vitamins, minerals, and phytochemicals are among the many nutrients and bioactive substances found in whole grain maize.*

 1. What about lutein and zeaxanthin? Major carotenoids in maize and important for human health but it was not mentioned.

 2. What about resistant starch?

**Response:**

 a. Lutein and zeaxanthin were mentioned under section 5 ( **Bioactive phytochemicals in Maize**) in page 14

 b. Resistant starch was not mentioned by any of the included paper to be a component of maize. This may be a reason why it is left out. The study reflected the components shown in the included papers. see page 36

**Comments 11:** *The nutraceutical potential of maize is a modern concern with maize. As a functional food, maize provides macronutrients along with bioactive compounds like polyphenols, carotenoids, phenolic acids, flavonoids, phytosterols, and lignins* – Delete polyphenols since specific polyphenol compounds are mentioned.

**Response:**

 That is being corrected

**Comments 12**

*Generally, the conventional variants of maize, amongst others, include iron, zinc, and vitamin A…* - Maize does not contain vitamin a but provitamin A compounds.

**Response:**

 This is being corrected and updated

**Comment 13**

*The lipids are present as trace elements…* - they are not elements but compounds.

**Response:**

 This has been updated too.

**Comment 14**

*These practices not only enhance macro- and micronutrient contents of the grain but also improve overall yield and quality.* – These practices can affect composition and yield, depending on the practice, and whether this effect is going to be positive. Rephrase the sentence. In addition, some of the practices were mentioned in the text (page 4) but they would better fit in this part.

**Response:**

 The sentence has been rephrased to reflect thus:

 These practices do not only boost the macro- and micronutrient contents of the grain, but they also contribute to improved yield and overall crop quality.
